# Angiopoietin-2, Angiopoietin-1 and subclinical cardiovascular disease in Chronic Kidney Disease

**DOI:** 10.1038/srep39400

**Published:** 2016-12-19

**Authors:** Yi-Chun Tsai, Chee-Siong Lee, Yi-Wen Chiu, Hung-Tien Kuo, Su-Chu Lee, Shang-Jyh Hwang, Mei-Chuan Kuo, Hung-Chun Chen

**Affiliations:** 1 Graduate Institute of Clinical Medicine, College of Medicine, Kaohsiung Medical University, Kaohsiung, Taiwan; 2 Division of General Medicine, Kaohsiung Medical University Hospital, Kaohsiung, Taiwan; 3 Division of Nephrology, Kaohsiung Medical University Hospital, Kaohsiung, Taiwan; 4 Faculty of Renal Care, Kaohsiung Medical University, Kaohsiung, Taiwan; 5 Division of Cardiology, Kaohsiung Medical University Hospital, Kaohsiung, Taiwan; 6 Institute of Population Sciences, National Health Research Institutes, Miaoli, Taiwan

## Abstract

Angiopoietins (Angpt) and vascular endothelial growth factor (VEGF) have been associated with cardiovascular disease. The study enrolled 270 pre-dialysis stage 3–5 CKD patients to assess the link between circulating Angpt2, Angpt1 and VEGF-A and subclinical measures of cardiovascular structure and function. Serum markers of angiogenesis were measured using commercial enzyme-linked immunosorbent assays. Cardiac structure and function were examined by echocardiography. Brachial-ankle pulse wave velocity (baPWV) was measured by the ankle-brachial index. The adjusted mean of left ventricular mass index (LVMI) was 2.05 in patients of Angpt2 quartile 4 and 1.99 in those of Angpt2 quartile 1 (P = 0.04). Angpt2 was significantly associated with LV hypertrophy (LVH) (Angpt2 quartile 4 compared with Angpt2 quartile 1: adjusted OR: 2.68, 95% CI: 1.15–6.20). Angpt1 was negatively correlated with left atrial diameter (adjusted mean of LAD: 3.59 in Angpt1 quartile 4, 3.92 in Angpt1 quartile 1, P = 0.03). A positive and significant correlation was found between Angpt2 level and baPWV in spearman’s correlation, but not in adjusted model. In conclusion, high Angpt2 and low Angpt1 levels were positively associated with abnormal cardiac structure in stages 3–5 CKD patients, which is compatible with the viewpoint that angiopoietins participates in cardiovascular burdens.

Angiopoietins, as one of the endothelial growth factors, modulates vascular development and remodeling during angiogenesis and inflammation process^1^. There are two major types of angiopoietins: angiopoietin-1 (Angpt1) and angiopoietin-2 (Angpt2), and both of them bind to the same endothelial receptor, Tie-2. Angpt1 binds to Tie2 receptor and then activates downstream signaling, thereby stabilizing endothelial and vascular structure. However, Angpt2 possesses opposite physiological properties and expressions of Angpt1. Angpt2 interrupts Ang-1-Tie-2 signaling, and then contributes to structural and functional changes of vessels through the effect of vascular endothelial grower factor (VEGF)^2^.

Accumulating evidence has demonstrated that high circulating Angpt2 and VEGF levels were shown in cardiovascular diseases, including congestive heart failure^3^ and coronary artery disease^4^. Elevated Angpt2 and VEGF levels were also significantly associated with traditional risk factors for cardiovascular diseases (CVD), such as blood pressure and metabolic syndrome^5^,^6^. Furthermore, a significant relationship between Ang-2 and cardiovascular mortality had been mentioned in general population^7^. Our previous report also found that high Angpt2 level was significantly associated with major adverse *cardiac events* (MACEs) in chronic kidney disease (CKD) not on dialysis. Angpt2 was as an independent predictor of cardiovascular burdens^8^.

CKD patients have higher risk of developing CVD and all-cause mortality^9^,^10^. Apart from the traditional risk factors, endothelial dysfunction has been shown to associates of cardiovascular morbidity and mortality^11^. In the study by David *et al*., Angpt2 was notably elevated in CKD patients either on dialysis or not^12^. Circulating Angpt2, not Angpt1 level was positively correlated with coronary artery disease and peripheral artery disease scores in dialysis and transplant patients^13^. The mechanisms mediating the increased cardiovascular burdens are not well-known. Shroff *et al*. indicated a significant association of Angpt2 with intima media thickness in children on dialysis^14^. Angpt2 was also correlated with ventricular dysfunction and the clinical stages of heart failure in congenital heart disease, but the consistent association was not related to Angpt1 and VEGF^15^. Based on limited results in CKD patients not on dialysis, this study aimed to analyze the association of serum markers of angiogenesis including Angpt2, Angpt1 and VEGF-A with subclinical measures of cardiovascular function and structure in patient with CKD stages 3–5.

## Results

### Characteristics of the Entire Cohort

The comparison of clinical characteristics between patients stratified by quartiles of circulating Angpt2 level, cut at 1538.2, 1990.7, and 2753.2 pg/ml is shown in Table 1. The study population consisted of 270 patients with a mean age of 65.4 ± 12.3 years and 56.7% of male. Among them, 87.4% were hypertensive, 41.1% were diabetes, and 21.9% had CVD. There was a significant difference of the proportion of diabetes and β-blocker usage, and serum blood urea nitrogen, estimated glomerular filtration rate (eGFR), hemoglobin, albumin and total calcium levels among Ang-2 quartiles. Serum calcium level was higher in CKD patients with Angpt2 quartile 3 than those with Angpt2 quartile 1. The proportion of usage of calcium channel blocker and β-blocker was the highest in CKD patients with Angpt2 quartile 4. Serum albumin and hemoglobin levels were lower in CKD patients with Angpt2 quartile 4 than those with Angpt2 quartile 1. Urine protein was higher in CKD patients with Angpt2 quartile 4 than other groups. Patients with Angpt2 quartile 4 had the highest incidence of CKD stage 5 and the lowest incidence of CKD stage 3 among 4 groups.

### Correlation between circulating Angpt2, Angpt1 and VEGF-A levels and cardiac structure and function

Table 2 shows the distribution of echocardiographic parameters in study patients stratified by Angpt2 quartiles. Of all patients, the median of left ventricular mass index (LVMI) was 133 g/m^2^ and 25.9% of those had concentric left ventricular hypertrophy (LVH) and 40.7% of those had eccentric LVH. The medians of left ventricular ejection fraction (LVEF) and left ventricular fraction shortening (LVFS) were 70.1 ± 10.2% and 40.6 ± 8.4% respectively. 73.9% of patients had peak early transmitral filling wave velocity (E)/peak late transmitral filling wave velocity (A) less than 1. There was a significant difference of LVMI and the proportion of LVH among Angpt2 quartiles (P = 0.04 and 0.02 respectively). LVMI and the proportion of LVH were higher in Angpt2 quartile 4 compared with Angpt2 quartile 1. CKD patients with Angpt2 quartile 4 had higher proportion of concentric LVH than those with Angpt2 quartile 1. The rate of eccentric LVH was higher in Angpt2 quartile 3 compared with Angpt2 quartile 1. However, there was no difference of LAD, LVEF, LVFS, and E/A ratio across Angpt2 quartiles.

**Figure 1:**
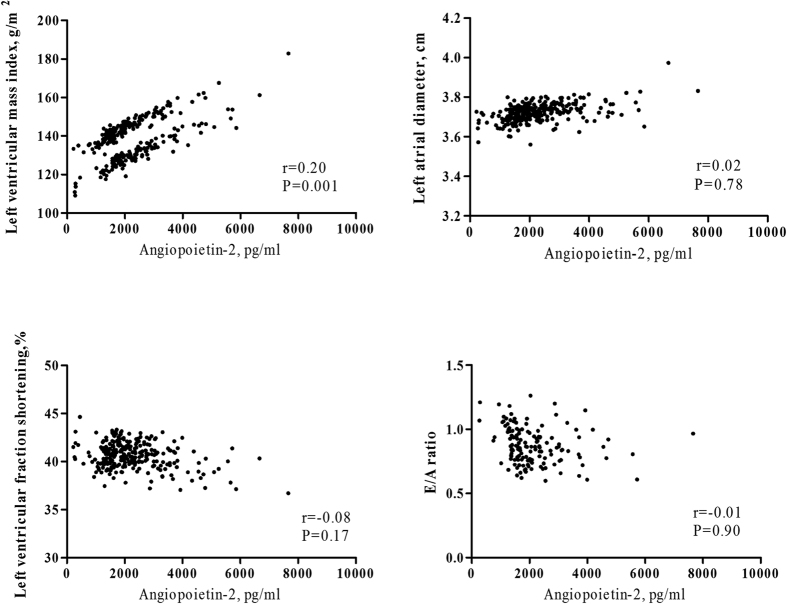
The age and sex-adjusted correlation between circulating angiopoietin-2 level and parameters of echocardiography.

No significant association of circulating Angpt2 level with LAD, LVFS, and E/A ratio was shown in age and sex-adjusted and multivariable-adjusted linear regression (Fig. 1 and Table 3). Circulating Angpt1 and Angpt2/Angpt1 levels were not correlated with LVFS and E/A ratio, except LAD. Angpt1 (β = −0.30, P = 0.02) and Angpt2/Angpt1 (β = 0.23, P = 0.03) were significantly correlated with LAD in age and sex-adjusted analysis. After adjustment of well-known factors, Angpt1, not Angpt2/Angpt1, was negatively associated with LAD in CKD patients (β = −0.30, P = 0.04). The adjusted mean of LAD of Angpt1 quartile 4 compared to quartile 1 was 3.59 cm and 3.92 cm (P = 0.03, Table 4). The significant correlation between stepwise increases in Angpt 1 levels and LAD was shown in patients with CKD stages 3–5 (P-trend = 0.04, Table 4).

Figure 1 shows a significant age and sex-adjusted correlation between circulating Angpt2 level and LVMI (r = 0.20, P = 0.001). The multivariable-adjusted unstandardized β of log-formed LVMI was also significant for every one increase in log-formed Angpt2 (β = 0.09, P = 0.03) and every one increase in log-formed Angpt2/Angpt1 (β = 0.05, P = 0.03, Table 3). The adjusted means of log-formed LVMI were higher in CKD patients with Angpt2 quartile 4 (2.05) than in those with Angpt2 quartile 1 (1.99, Table 5). The significant correlation between stepwise increases in Angpt2 levels and LVMI was found in patients with CKD stages 3–5 (P-trend = 0.03). The unadjusted and adjusted odds ratios (OR) of LVH were 3.63 (95% CI: 1.71–7.71) and 2.68 (95% CI: 1.15–6.20) for patients of Angpt2 quartile 4 compared with those of Angpt2 quartile 1. There was significant association of stepwise increases in Angpt2 levels with LVH (P-trend  = 0.03, Table 6). However, the significant association of LVMI with Angpt1 or VEGF-A was not shown in adjusted linear regression analysis (Table 3).

Furthermore, we performed subgroup analysis and stratified CKD patients by high-sensitivity C-reactive protein (hsCRP) of 1 mg/L and eGFR of 20 ml/min/1.73 m^2^ respectively. High circulating Angpt2 level was still significantly associated with LVH in CKD patients with hsCRP ≦ 1 mg/L (P-trend = 0.02) and those with eGFR ≧ 20 ml/min/1.73 m^2^ (P-trend = 0.007) after adjustment of age and sex, but not in those with hsCRP >1 mg/L and those with eGFR < 20 ml/min/1.73 m^2^.

### Correlation between circulating Angpt2, Angpt1 and VEGF-A levels and brachial-ankle pulse wave velocity (baPWV)

**Figure 2:**
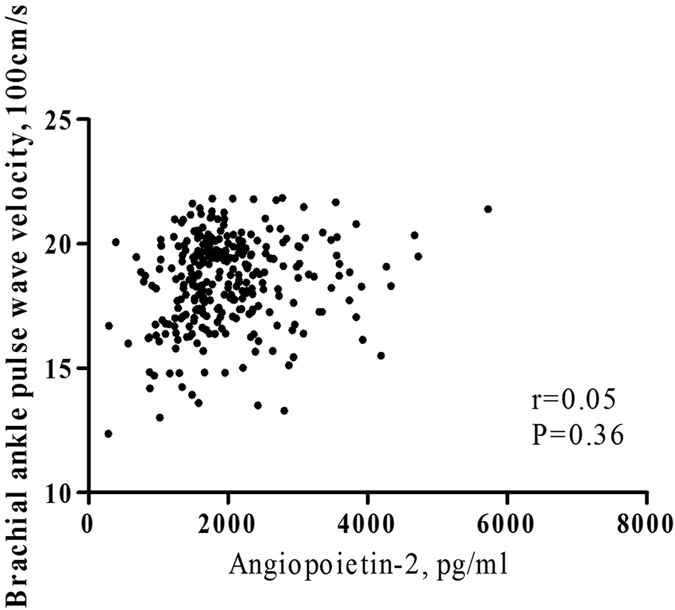
The age and sex-adjusted correlation between circulating angiopoietin-2 level and brachial ankle pulse wave velocity.

A positive and significant correlation was found between baPWV and circulating Angpt2 level (spearman r = 0.20, P = 0.02). Besides, baPWV was significantly correlated with Angpt1 (spearman r = −0.27, P = 0.01) and Angpt2/Angpt1 (spearman r = 0.28, P = 0.007), but not with VEGF-A. However, there was no significant association of baPWV with Angpt2, Angpt1 or Angpt2/Angpt1 in adjusted analysis (Fig. 2 and Table 3).

## Discussion

This study assessed the relationship between serum markers of angiogenesis including Angpt2, Angpt1, and VEGF-A and measures of cardiovascular structure and function as subclinical features of CVD in CKD patients not on dialysis. The results show that high circulating Angpt2 level was significantly associated with the increase of LVMI and LVH and the negative correlation between circulating Angpt1 level and LAD was also shown in patients with stages 3–5 CKD after adjustment for traditional cardiovascular risk factors. There was no significant association of Angpt2, Angpt1, and VEGF-A with either LVFS or E/A ratio. It meant that serum markers of angiogenesis were not associated with LV systolic or diastolic function in CKD patients.

Circulating Angpt2 level has been a potential predictor of the accelerated development of cardiovascular burdens^8^,^13^. Overexpression of Ang-2 leaded to endothelial apoptosis, and promoted of expression of adhesion molecules, such as intercellular adhesion molecule 1 (ICAM-1) and vascular cell adhesion molecule 1 (*VCAM*-*1*), thereby playing a crucial role in the progression of cardiac hypertrophy and interstitial fibrosis in animal model^16^. Lee *et al*. found these patients had higher Angpt2 level than control group and further observed a serial changes in circulating Angpt2 level from the acute to the chronic stages in myocardial infarction^17^. Eleuteri *et al*. investigated the correlation between Angpt2 and the index of cardiac function impairment and showed that Angpt2 level was significantly correlated with the severity of congestive heart failure^18^. The study of Health in Pomerania (SHIP) that included 3204 normal subjects with a median age of 55 years to evaluate the relationship between Ang-2 and cardiac function revealed that high circulating Angpt2 level was associated with low LVFS value as a measure of LV systolic function, but not with LVMI as a measure of LV structure^19^. However, the information between Angpt2 and associated markers of angiogenesis and actual cardiac structure and function status is limited in CKD population.

This present study shows that high circulating Angpt2 level, not Angpt1 or VEGF-A was significantly associated with high LVMI level and increased risk for LVH, but not with either LV systolic or diastolic function in CKD patients, whereas these findings were inconsistent with those of SHIP in general population. Comparing with SHIP, our participants were older, had lower baseline eGFR, higher proportion of diabetes and anti-hypertensive drugs usage. A large dissimilarity of the participants may partially explain the inconsistency of results between the two studies. Structural and functional impairment of heart is frequently mentioned in CKD patients and these patients are easier to have high burdens of cardiovascular morbidity and mortality. There was no big difference of LVMI across Angpt2 quartiles 1–3 in our CKD patients. Patients with Angp2 quartile 4 had the lowest eGFR and highest serum hsCRP level than others. Angpt2, impaired renal function, and inflammation might have a synergic effect on cardiac hypertrophy. The significant association between Angpt2 and LVH was only consistent in patients with eGFR ≧ 20 ml/min/1.73 m^2^. Thus, further studies are needed to explore the mechanisms of the interaction between Angpt2, renal function and cardiac structure.

Chronic inflammation has been considered as a crucial factor in atherosclerosis development. Masiha *et al*. reported that inflammatory markers such as high-sensitivity c –reactive protein (hsCRP), ICAM-1, and VCAM-1 were elevated in concentric LVH, and hsCRP was correlated with LV diastolic function, while inflammation might play a pathogenetic role in abnormal LV geometry and function^20^. On the other hand, Angpt2 is associated with pro-inflammatory responses with P-selectin, E-selectin and VCAM-1^12^. Angpt2 triggers the infiltration of neutrophils and monocytes and Angpt2-induced signaling leads to induction of adhesion molecules and vascular leakage^21^. It is possible that the influence of the interaction between inflammation and Angpt2 on cardiac function and structure exists. Our subgroup analysis found that circulating Angpt2 level was significantly associated with LVH in CKD patients with hsCRP ≦ 1 mg/L not in those with hsCRP>1 mg/L. There might be a complicated interaction between Angpt2 and inflammation in cardiac remodeling.

The Angpt-Tie system has been considered as a major regulator of vascular and endothelial cell maintenance. Vascular and endothelial dysfunction would contribute to cardiovascular morbidity and mortality. The study conducted by Chang *et al*.^22^ supported that Angpt2 was positively associated with baPWV, a clinical marker of arterial stiffness, in CKD patients not on dialysis. However, the significant linear correlation between Angpt2 and baPWV was not consistent in our CKD patients after adjusting well-known variables. The present results show that baPWV seemed to increase from Angpt2 quartile 1 to 3, but decreased in Angpt2 quartile 4. We supposed that poor renal function and inflammation might interfere in the association of Angpt2 with baPWV. Additionally, David *et al*. has speculated a better relationship between Angpt2 and coronary artery disease than those between Angpt2 and peripheral artery disease in patients with CKD stage 4 and dialysis^23^. The dissimilarity is probable that the alterations in the Ang/Tie axis might be different between coronary arterial endothelium and peripheral arterial endothelium^24^. Further study is needed to investigate the mechanism of association between Ang-2 and the different vascular beds for these inconsistent results.

Among angiopoietin families, Angpt2 had been more strongly related to increased risks for cardiovascular burdens than Angpt1 in previous clinical reports^3^,^4^,^13^. The information of Angpt1 in subclinical cardiovascular disease is limited. We demonstrated the novel finding of a negative association between Angpt1 and LAD, as a predictor of adverse cardiovascular outcome in CKD patients^25^. The balance between Angpt1 and Angpt2 regulates vascular development and remodeling^1^. Angpt1 could restrain inflammatory process and abnormal angiogenesis^1^. Decreased Angpt1 might have a potential influence on the change of cardiac structure. Conversely, there was no significant correlation between VEGF-A and cardiovascular structure in our CKD cohort. Nevertheless, VEGF-A still has played a role in modulation of Angpt2-mediated signaling in pathophysiology.

Some limitations must be considered in this study. Firstly, serum markers of angiogenesis and echocardiography parameters were measured once at enrollment. The effect of the time-varying serum markers of angiogenesis levels on cardiac structure and function might be underestimated. Although we found the significant association of Angpt2 with cardiac hypertrophy and correlation between Angpt1 and LAD, the p-values for trend seemed to be low. The relative small sample size probably diminished the statistic power. Nevertheless, Angpt2 and Angpt1 seemed to be a potential marker of cardiac remodeling.

In conclusion, our study demonstrates that high circulating Angpt2 is associated with increased risks for LVH and Angpt1 was negatively correlated with LAD in stages 3–5 CKD patients. These findings promote the notion that Angpt2 and Angpt1were potential biomarkers of cardiac remodeling in CKD patients. Future studies will be necessary to investigate the pathogenic role of Angpt2 and Angpt1 in cardiac structure and function.

## Methods

### Study Samples

This observational study enrolled 270 CKD stages 3–5 patients, who had been enrolled in our integrated CKD program for more than 3 months. These patients were scheduled for a study interview after they have given and written informed consent between 2006 and 2011 at Kaohsiung Medical University Hospital in Southern Taiwan. CKD was staged based on Kidney Disease Outcomes Quality Initiative (K/DOQI) guidelines. The eGFR was calculated according to Levey *et al*.^26^. This study protocol was approved by the Institutional Review Board of the Kaohsiung Medical University Hospital (KMUH-IRB-990198)^8^. The methods were carried out in accordance with the relevant guidelines, including any relevant details.

### Clinical measurements

Information on socio-demographic characteristics and clinical data including age, gender, cigarette smoking, alcohol, medication, and co-morbidities were obtained by medical records and interviews with patients at enrollment. Diabetes was defined by self-report, use of anti-diabetic drugs, or blood glucose values according to the American Diabetes Association criteria. Hypertension was defined as patients with a history of hypertension, taking antihypertensive drugs, or the measurement of blood pressure ≧140/90 mmHg. Heart disease was defined as a history of myocardial infarction, ischemic heart disease or congestive heart failure. Information regarding patients’ medications, such as angiotensin converting enzyme inhibitors (ACEI), angiotensin II receptor blockers (ARB), β-blockers, and calcium channel blockers before and after enrollment was collected from medical records. Blood pressure was measured in the seated position after 5 min rest, using one single calibrated device. Blood pressure was recorded as the mean of three consecutive measurements with 5 minutes interval.

### Laboratory measurements

Fasting blood samples were collected at enrollment for the biochemistry study, including hemoglobin, glycated hemoglobin, albumin, phosphate, calcium, uric acid, cholesterol, triglyceride, hsCRP, and parathyroid hormone. The markers of angiogenesis including Angpt1, Angpt2, and VEGF-A levels were measured using commercial enzyme-linked immunosorbent assays (R&D Systems Inc). The sensitivity of Angpt1, Angpt2, and VEGF-A assay was 10.3, 1.20, and 5 pg/ml. The interassay and intraassay coefficients of variation of Angpt2 were 1.2% and 1.8%. The interassay and intraassay coefficients of variation of Angpt1 were 4.3% and 5.1%. The interassay and intraassay coefficients of variation of VEGF-A were 6.3% and 5.3%. All assays were assessed in duplicate by investigators blinded to the characteristics and clinical outcome of patients.

### Evaluation of cardiac structure and function

The echocardiographic examination was performed at the same time as blood and urine sample collection by experienced cardiologists using VIVID 7 (General Electric Medical Systems, Horten, Norway), while patients breathed quietly in the left decubitus position. The cardiologists were blind to the clinical characteristics and laboratory data of patients. Two-dimensional and M-mode images were recorded from the standardized views. The echocardiographic measurements including LAD, AoR, left ventricular posterior wall thickness in diastole and systole (LVPWTd and LVPWTs), left ventricular internal diameter in diastole and systole (LVIDd and LVIDs), interventricular septal wall thickness in diastole and systole (IVSd and IVDs), E, and A were obtained. E/A ratio less than one was defined as diastolic dysfunction. Left ventricular systolic function was assessed according to LVFS and LVEF. In addition, left ventricular mass (LVM) was calculated using Devereux-modified method^27^, while LVMI was calculated by dividing left ventricular mass by body surface area. The definition of LVH was based on the 2007 European Society of Hypertension/European Society of Cardiology guidelines^28^. Left ventricular relative wall thickness (LVRWT) was calculated as the ratio of 2 × LVPWTd/LVIDd. Concentric LVH was defined as LVMI ≧ 125 g/m^2^ in men and ≧110 g/m^2^ in women, with LVRWT ≧ 0.45; eccentric LVH was defined as LVMI ≧ 125 g/m^2^ in men and ≧110 g/m^2^ in women, with LVRWT < 0.45. Stroke volume was calculated using measurements of ventricle volumes and subtracting the volume of the blood in the ventricle at the end of a beat (end-systolic volume) from the volume of blood just prior to the beat (end-diastolic volume). Based on the unmeasured E/A ratio in atrial fibrillation, this study excluded CKD patients with atrial fibrillation.

### Assessment of brachial-ankle pulse wave velocity

BaPWV, as a fine parameter of arterial stiffness, was measured by the ankle-brachial index (ABI)-form device (Colin VP1000, Komaki, Japan) at enrollment. This device automatically and simultaneously measured blood pressures in both arms and ankles using an oscillometric method^29^,^30^. Pulse waves obtained from the brachial and tibial arteries were recorded simultaneously, and the transmission time (ΔTba), which was defined as the time interval between the initial increase in brachial and ankle waveforms, was determined for baPWV measurement. The transmission distance from the brachium to ankle was calculated according to body height. The path length from the suprasternal notch to the brachium(Lb) was obtained using the following equation: Lb = 0.2195 × height of the patient (in cm) − 2.0734. The path length from the suprasternal notch to the ankle (La) was obtained using the following equation: La = (0.8129 × height of the patient [in cm] +12.328). Finally, the following equation was used to automatically obtain baPWV: baPWV = (La − Lb)/ΔTba. The higher baPWV value was used to represent arterial stiffness for each patient.

### Statistical Analysis

Statistical results of baseline characteristics of all patients were stratified by quartiles of Angpt2, with cutoff values of 1538.2, 1990.7, and 2753.2 pg/ml respectively based on the non-normal distribution of Angpt2. Continuous variables were expressed as mean ± SD or median (25^th^, 75^th^ percentile) as appropriate. Skewed distribution of continuous variables, such as Angpt2, Angpt1, VEGF and LVMI was log-transformed to approximate normal distribution. Categorical variables were expressed as percentages. The differences in continuous variables between groups were evaluated by one-way analysis of variance (ANOVA) or the Kruskal-Wallis H test as appropriate. Bonferroni post hoc analysis was also used for evaluation between two groups after ANOVA or the Kruskal-Wallis H test. The differences in categorical variables between groups were tested by Chi-square test. Furthermore, the Spearman correlation and adjusted partial correlation were performed to analyze the correlation between serum markers of angiogenesis, baPWV and echocardiography parameters. Univariate and multivariate linear regression and logistic regression were utilized to investigate the association between serum markers of angiogenesis and echocardiography parameters. Adjusted variables included age, sex, smoking history, diabetes mellitus, heart disease, eGFR, and serum hemoglobin and cholesterol levels. Statistical analyses were conducted using SPSS 18.0 for Windows (SPSS Inc., Chicago, Illinois). Statistical significance was set at a two-sided p-value of less than 0.05.

## Additional Information

**How to cite this article**: Tsai, Y.-C. *et al*. Angiopoietin-2, Angiopoietin-1 and subclinical cardiovascular disease in Chronic Kidney Disease. *Sci. Rep.* **6**, 39400; doi: 10.1038/srep39400 (2016).

**Publisher's note:** Springer Nature remains neutral with regard to jurisdictional claims in published maps and institutional affiliations.

## Figures and Tables

**Table 1:** The clinical characteristics of study subjects stratified by circulating angiopoietin-2 quartile.

	Entire Cohort N = 270	Angiopoietin-2^a^				
		Quartile 1 (≦1538.2 pg/ml) N = 67	Quartile 2 (>1538.2, ≦1990.7 pg/ml) N = 68	Quartile 3 (>1990.7, ≦2753.2 pg/ml) N = 68	Quartile 4 (>2753.2 pg/ml) N = 67	P-value^b^
Demographics						
Age, year	65.4 ± 12.3	61.3 ± 12.8^#†^	67.8 ± 10.4^*^	67.6 ± 10.7^*^	64.8 ± 14.2	0.006
Sex (male), %	153(56.7)	50(74.6)	35(51.5)	38(55.9)	30(44.8)	0.004
Smoke, %	50(18.5)	16(23.9)	8(11.8)	12(17.6)	14(20.9)	0.30
Alcohol, %	23(8.5)	8(11.9)	8(11.8)	2(2.9)	5(7.5)	0.19
Cardiovascular disease, %	59(21.9)	11(16.4)	15(22.1)	17(25.0)	16(23.9)	0.63
Hypertension, %	236(87.4)	62(92.5)	58(85.3)	57(83.8)	59(88.1)	0.44
Diabetes mellitus, %	111(41.1)	23(34.3)	25(36.8)	26(38.2)	37(55.2)	0.06
CKD stage 3, %	39(14.4)	11(16.4)	10(14.7)	12(17.6)	6(9.0)^#^	0.03
4, %	107(39.6)	29(43.3)	35(51.5)	24(35.3)	19(28.4)^#^	
5, %	124(45.9)	27(40.3)	23(33.8)	32(47.1)	42(62.7)^#^	
Body Mass Index, kg/m^2^	24.9 ± 4.0	24.1 ± 3.6^#^	25.6 ± 3.7^*^	24.4 ± 3.3	25.5 ± 5.1	0.06
Systolic blood pressure, mmHg	137 ± 19	134 ± 14^#^	136 ± 18^*^	138 ± 20	144 ± 22	0.22
Diastolic blood pressure, mmHg	75 ± 12	79 ± 11^#^	73 ± 12^*^	74 ± 12	73 ± 12	0.15
Pulse wave velocity, 100 cm/s	18.5 ± 3.5	17.5 ± 3.6	18.9 ± 4.0	19.3 ± 2.6	18.2 ± 3.3	0.17
Medication						
Calcium channel blocker (%)	159(58.9)	38(56.7)	43(63.2)	30(44.1)	48(71.6)	0.01
β-blocker (%)	70(25.9)	12(17.9)	14(20.6)	18(26.5)	26(38.8)	0.03
ACEI/ARB (%)	156(57.8)	38(56.7)	44(64.7)	37(54.4)	37(55.2)	0.60
Laboratory parameters						
Blood urea nitrogen (mg/dl)	47.2(35.3,65.2)	46.6(32.5,64.9)	40.5 (31.2,52.8)	46.9(36.5,63.7)	57.3(46.1,74.1)^*#†^	<0.001
eGFR (ml/min/1.73 m^2^)	18.7 ± 10.8	19.9 ± 11.6	20.8 ± 9.9	18.7 ± 11.5	15.2 ± 9.2^#^	0.01
Glycated hemoglobin (%)	5.9(5.5,6.8)	5.8(5.5,6.6)	5.9(5.5,6.6)	5.9(5.5,6.3)	6.3(5.4,7.7)	0.54
Hemoglobin (g/dl)	10.8 ± 2.0	11.5 ± 1.9^†^	11.1 ± 2.0	10.6 ± 2.0^*^	10.0 ± 1.7^*#^	<0.001
Albumin (g/dl)	4.0 ± 0.5	4.2 ± 0.4	4.0 ± 0.4	4.0 ± 0.4	3.9 ± 0.5^*^	0.02
Phosphate (mg/dl)	4.2(3.7,5.0)	4.1(3.6,4.8)	4.0(3.6,4.7)	4.2(3.7,4.8)	4.5(4.0,5.2)	0.02
Calcium (mg/dl)	8.9 ± 0.7	9.1 ± 0.6^†^	9.0 ± 0.7	8.7 ± 0.6^*^	8.9 ± 0.8	0.03
Uric acid (mg/dl)	7.8 ± 2.0	7.9 ± 2.0	7.6 ± 1.8	7.5 ± 1.7	8.1 ± 2.2	0.32
Cholesterol (mg/dl)	188 ± 46	193 ± 51	190 ± 37	175 ± 44	192 ± 47	0.08
Triglyceride (mg/dl)	116(77,169)	99(74,170)	112(83,160)	113(75,149)	127(84,186)	0.50
hsCRP (mg/L)	1.5(0.6,4.2)	1.4(0.5,4.2)	1.5(0.6,3.7)	1.3(0.6,3.4)	1.9(0.5,6.9)	0.67
Urine protein-creatinine ratio (mg/mg)	1.2(0.5,2.5)	1.0(0.5,1.8)	1.1(0.4,1.8)	0.9(0.5,2.1)	1.8(0.9,3.2)^*#†^	0.005
Angiopoietin-2 (pg/ml)	1983(1537,2749)	1320(1072,1405)	1734(1623,1889)^*†^	2335(2145,2501)^*#^	3548(3034,4390)^*#†^	<0.001
Angiopoietin-1 (pg/ml)	11378(5948,23148)	16243(7217,24244)	12817(5958,27255)	8778(3510,16812)	10573(5920,20440)	0.06
VEGF-A (pg/ml)	56.4(39.3,81.1)	61.2(39.2,85.3)	53.6(42.2,79.8)	47.5(36.8,66.1)	67.8(40.0,97.1)	0.04

Data are expressed as number (percentage) for categorical variables and mean ± SD or median (25^th^, 75^th^ percentile) for continuous variables, as appropriate.

Abbreviations: CKD, chronic kidney disease; ACEI, angiotensin converting enzyme inhibitors; ARB, angiotensin II receptor blockers; eGFR, estimated glomerular filtration rate; hsCRP, high-sensitivity C-reactive protein.

Bonferroni post hoc analysis was utilized for analysis between two groups.

^*^P < 0.05 compared with quartile 1; ^#^*P* < 0.05 compared with quartile 2; ^†^*P* < 0.05 compared with quartile 3.

^a^Angiopoietin-2 quartile cut at 1538.2, 1990.7, and 2753.2 pg/ml.

^b^P-value calculated from one-way analysis of variance (ANOVA) test.

**Table 2:** The echocardiographic parameters of study patients stratified by circulating angiopoietin-2 quartile.

	Entire Cohort N = 270	Angiopoietin-2^a^				
		Quartile 1 (≦1538.2 pg/ml) N = 67	Quartile 2 (>1538.2, ≦1990.7 pg/ml) N = 68	Quartile 3 (>1990.7, ≦2753.2 pg/ml) N = 68	Quartile 4 (>2753.2 pg/ml) N = 67	P-value^b^
Aortic root diameter, cm	3.2 ± 0.4	3.2 ± 0.5	3.3 ± 0.5	3.2 ± 0.4	3.2 ± 0.4	0.27
Left atrium diameter, cm	3.7 ± 0.7	3.6 ± 0.7	3.8 ± 0.6	3.8 ± 0.8	3.8 ± 0.7	0.37
Left atrium diameter/Aortic root diameter	1.2 ± 0.3	1.1 ± 0.3	1.2 ± 0.3	1.2 ± 0.3	1.2 ± 0.3	0.32
LVMI, g/m^2^	133(107,164)	126(95,150)	130(106,155)	131(110,165)	152(116,187)^*^	0.04
LVH, n(%)	180(66.7)	37(55.2)	42(61.8)	48(70.6)	53(79.1)^*^	0.02
LV geometry						0.02
Non-LVH, n(%)	90(33.3)	30(44.8)	26(38.2)	20(29.4)	14(20.9)^*^	
Concentric LVH, n(%)	70(25.9)	16(23.9)	16(23.5)	14(20.6)	24(35.8)^*^	
Eccentric LVH, n(%)	110(40.7)	21(31.3)	26(38.2)	34(50.0)^*^	29(43.3)^*^	
SV, ml	82.7 ± 24.7	79.7 ± 20.6	78.9 ± 20.6	86.0 ± 24.4	87.8 ± 32.6	0.23
LVEF, %	70.1 ± 10.2	70.9 ± 9.9	71.8 ± 10.5	67.8 ± 11.3	69.9 ± 8.6	0.15
LVFS, %	40.6 ± 8.4	41.4 ± 8.7	41.9 ± 8.38	39.1 ± 9.1	39.9 ± 7.1	0.19
E/A < 1, n(%)	105(73.9)	25(64.1)	31(79.5)	25(71.4)	24(82.8)	0.27

Data are expressed as number (percentage) for categorical variables and mean ± SD or median (25^th^, 75^th^ percentile) for continuous variables, as appropriate.

Abbreviations: LVMI, left ventricular mass index; LVH, left ventricular hypertrophy; SV, stroke volume; LVEF, left ventricular ejection fraction; LVFS, Left ventricular fraction shortening; E/A, peak early transmitral filling wave velocity/peak late transmitral filling wave velocity Bonferroni post hoc analysis was utilized for analysis between two groups.

^*^P < 0.05 compared with quartile 1; ^#^*P* < 0.05 compared with quartile 2; ^†^*P* < 0.05 compared with quartile 3.

^a^Angiopoietin-2 quartile cut at 1538.2, 1990.7, and 2753.2 pg/ml.

^b^P-value calculated from one-way analysis of variance (ANOVA) test.

**Table 3:** Relation of log-formed serum markers of angiogenesis with brachial ankle pulse wave velocity (baPWV) and measures of cardiac structure and function in CKD stages 3–5 subjects.

	Angpt2	Angpt1	Angpt2/Angpt1	VEGF
baPWV, per 100 cm/s Unstandardized β(95% Cl)				
Age and sex adjusted	1.07(−1.64,3.78)	−0.49(−2.14,1.15)	0.64(−0.67,1.94)	0.46(−2.96,3.88)
Multivariable adjusted	0.07(−2.03,2.17)	0.46(−1.58,2.49)	1.31(−0.67,3.28)	0.31(−3.76,4.36)
Log LVMI Unstandardized β(95% Cl)				
Age and sex adjusted	0.09(0.02,0.17)^a^	−0.04(−0.09,0.01)	0.05(0.01,0.09)^a^	−0.06(−0.14,0.02)
Multivariable adjusted	0.09(0.01,0.16)^a^	−0.02(−0.08,0.03)	0.05(0.00,0.09)^a^	−0.05(−0.14,0.04)
LAD, per 1 cm Unstandardized β(95% Cl)				
Age and sex adjusted	0.19(−0.18,0.57)	−0.30(−0.56, −0.04)^a^	0.23(0.02,0.44)^a^	−0.33(−0.75,0.08)
Multivariable adjusted	0.05(−0.36,0.45)	−0.30(−0.58, −0.02)^a^	0.18(−0.06,0.41)	−0.23(−0.67,0.21)
LVFS, per 1% Unstandardized β(95% Cl)				
Age and sex adjusted	−1.96(−6.42,2.51)	−0.28(−3.42,2.86)	−0.57(−3.11,1.96)	1.68(−3.28,6.65)
Multivariable adjusted	−1.01(−5.81,3.79)	−1.00(−4.48,2.47)	−0.11(−3.02,2.80)	0.52(−4.82,5.85)
E/A < 1 OR(95% CI)				
Age and sex adjusted	1.21(0.17,8.83)	0.38(0.10,1.52)	2.53(0.83,7.72)	5.34(0.51,56.30)
Multivariable adjusted	1.64(0.14,19.35)	0.41(0.07,2.32)	3.26(0.70,15.20)	7.77(0.49,123.13)

Abbreviations: LAD, left atrial diameter; LVFS, left ventricular fraction shortening; E/A, peak early transmitral filling wave velocity/peak late transmitral filling wave velocity; Angiopoietin-2, Angpt2; Angiopoietin-1, Angpt1; VEGF, vascular endothelial growth factor; OR, odds ratio.

Multivariable model: adjusted for age, sex, diabetes mellitus, heart disease, cigarette smoking, estimated glomerular filtration rate, serum hemoglobin and cholesterol levels.

^a^P-value < 0.05.

**Table 4:** Relation of circulating Angiopoietin-1 level with left atrial diameter (LAD) in CKD stages 3–5 subjects.

Adjusted mean of LAD (cm)	Angiopoietin-1				
	Quartile 1	Quartile 2	Quartile 3	Quartile 4	P-trend
Unadjusted model	3.95	3.62^b^	3.65^b^	3.56^a^	0.01
Age and sex adjusted model	3.94	3.62^b^	3.65^b^	3.57^b^	0.02
multivariate adjusted model	3.92	3.68	3.64	3.59^b^	0.04

Multivariate adjusted model: adjusted for age, sex, diabetes mellitus, heart disease, cigarette smoking, estimated glomerular filtration rate, serum hemoglobin and cholesterol levels.

^a^P-value < 0.01 compared wih quartile 1, ^b^P-value < 0.05 compared with quartile 1.

Angiopoietin-1 quartile cut at 5948.9, 11378.6 and 23148.7 pg/ml.

**Table 5:** Relation of circulating Angiopoietin-2 level with left ventricle mass index(LVMI) in CKD stages 3-5 subjects.

Adjusted mean of log-formed LVMI	Angiopoietin-2				
	Quartile 1	Quartile 2	Quartile 3	Quartile 4	P-trend
Unadjusted model	2.09	2.11	2.12	2.16^a^	0.006
Age and sex adjusted model	2.08	2.10^-^	2.12	2.16^a^	0.001
multivariate adjusted model	1.99	2.01	2.02	2.05^b^	0.04

Multivariate adjusted model: adjusted for age, sex, diabetes mellitus, heart disease, cigarette smoking, estimated glomerular filtration rate, serum hemoglobin and cholesterol levels.

^a^P-value < 0.01 compared wih quartile 1, ^b^P-value < 0.05 compared with quartile 1.

Angiopoietin-2 quartile cut at 1538.2, 1990.7, and 2753.2 pg/ml.

**Table 6:** Relation of circulating Angiopoietin-2 level with left ventricle hypertrophy (LVH) in CKD stages 3–5 subjects.

	Angiopoietin-2				
	Quartile 1	Quartile 2	Quartile 3	Quartile 4	P-trend
Odds ratio (95% Cl)					
Unadjusted model	Reference	1.31(0.66,2.60)	1.95(0.96,3.95)	3.07(1.43,6.56)^a^	0.002
Age and sex adjusted model	Reference	1.29(0.63,2.64)	1.91(0.91,3.98)	3.23(1.47,7.11)^a^	0.002
Multivariate adjusted model	Reference	1.33(0.62,2.82)	1.61(0.73,3.52)	2.36(1.02,5.49)^b^	0.04

Multuvariate adjusted model: adjusted for age, sex, diabetes mellitus, heart disease, cigarette smoking, estimated glomerular filtration rate, serum hemoglobin and cholesterol levels.

^a^P-value < 0.01 compared with quartile 1, ^b^P-value < 0.05 compared with quartile 1.

Angiopoietin-2 quartile cut at 1538.2, 1990.7, and 2753.2 pg/ml.
